# Supplementary material for: Effect of Nitrogen Application Rate on the Relationships between Multidimensional Plant Diversity and Ecosystem Production in a Temperate Steppe
Source: Biology (Basel). 2024 Jul 23;13(8):554. doi: 10.3390/biology13080554 (PMC11351205; doi:10.3390/biology13080554)
Supplement: Supplementary file 1 [file biology-13-00554-s001.zip › biology-3064450-supplementary.pdf]

Supplementary materials

Effect of nitrogen addition rate on the relationships between multi-dimensional plant diversity and ecosystem production in a temperate steppe

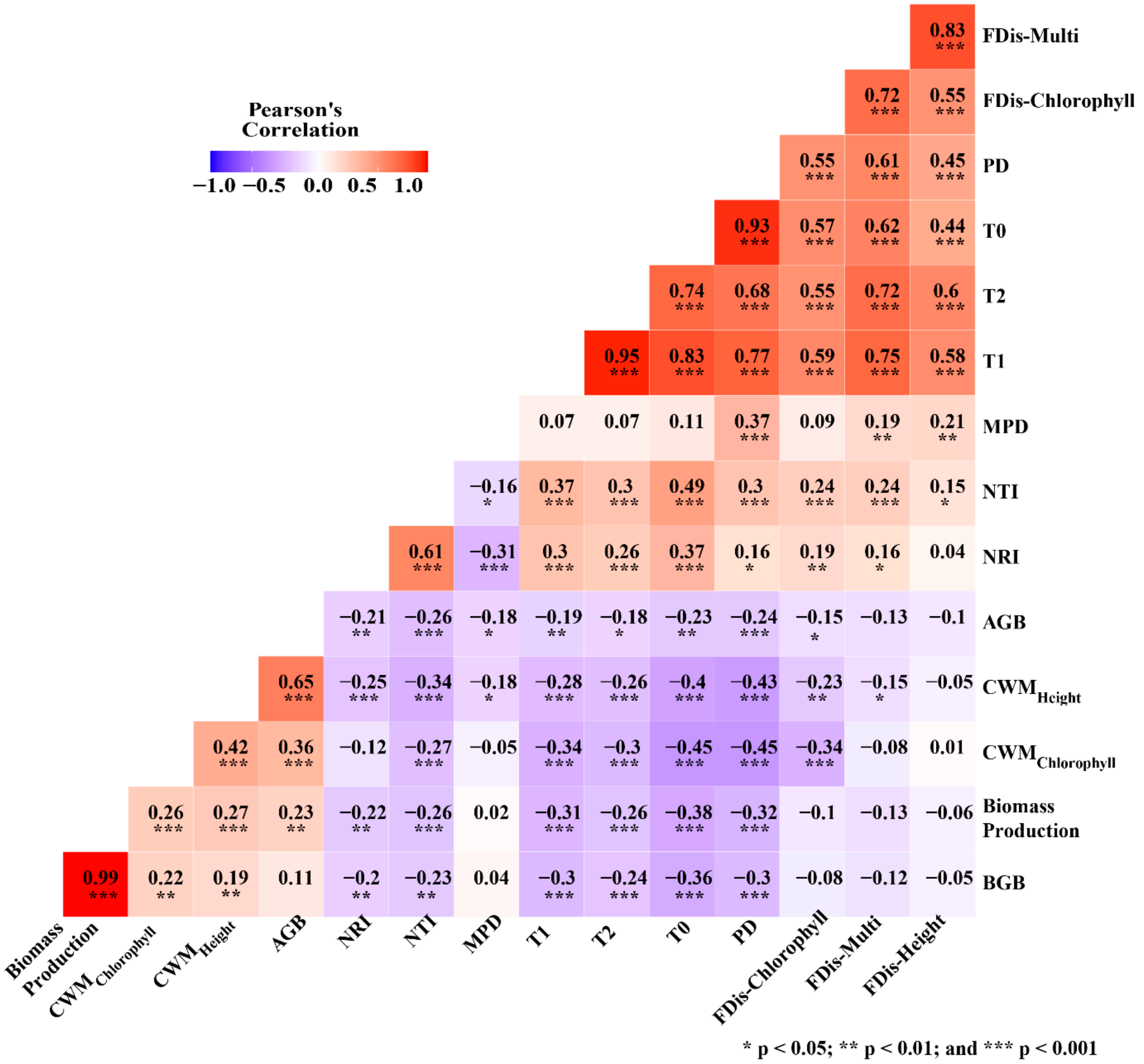

Figure S1: Result of Pearson's correlation analysis between plant diversity metrics and biomass production (values in the square box denotes correlation coefficients). T0: Species Richness (SR); T1: Shannon's diversity for taxonomic diversity index calculated using hill numbers (q=1); T2: inverse Simpson diversity index calculated using hill numbers (q=2); FDis-Multi: functional dispersion across two traits (i.e. Height and Chlorophyll content, describing the mean distance in multidimensional trait space of individual species to the centroid of all species; FDis-Height and FDis-Chlorophyll: Functional diversity of Height and Chlorophyll content, respectively; CWM_{Height} and $CWM_{Chlorophyll}$: Community-weighted mean of plant height and leaf chlorophyll content, values weighted by species abundance within the community, respectively; PD: Faith's community phylogenetic distance; MPD: Mean pairwise phylogenetic distance; NRI: Net

Relatedness Index; NTI: Net Taxonomic Index; AGB: plant Aboveground biomass, BGB: plant belowground biomass, Biomass Production: The sum of aboveground and belowground biomass used as surrogate for ecosystem biomass production. *, ** and *** represent $P < 0.05$, $P < 0.01$ and $P < 0.001$, respectively.

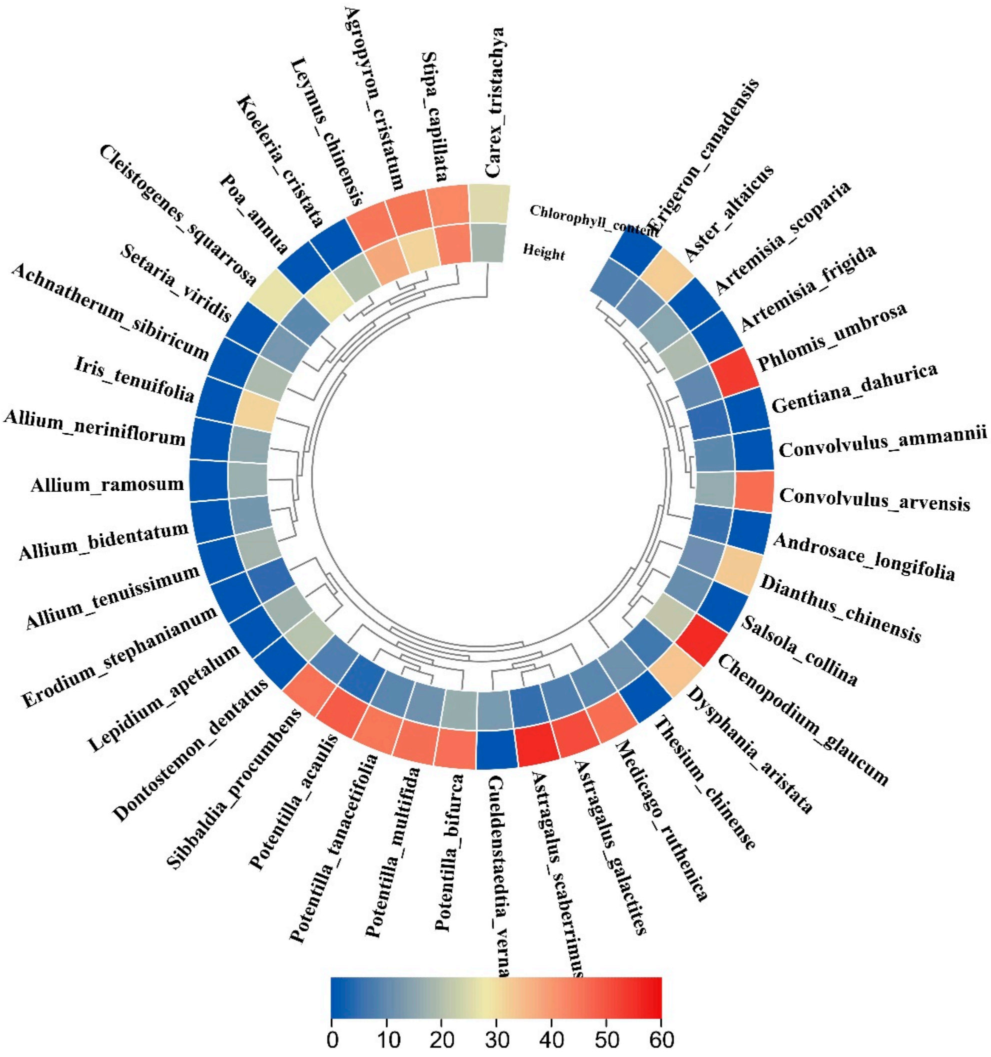

Figure S2: Phylogenetic tree of the plant species recorded in the experimental plots comprised of 40 tips and 39 internal nodes.

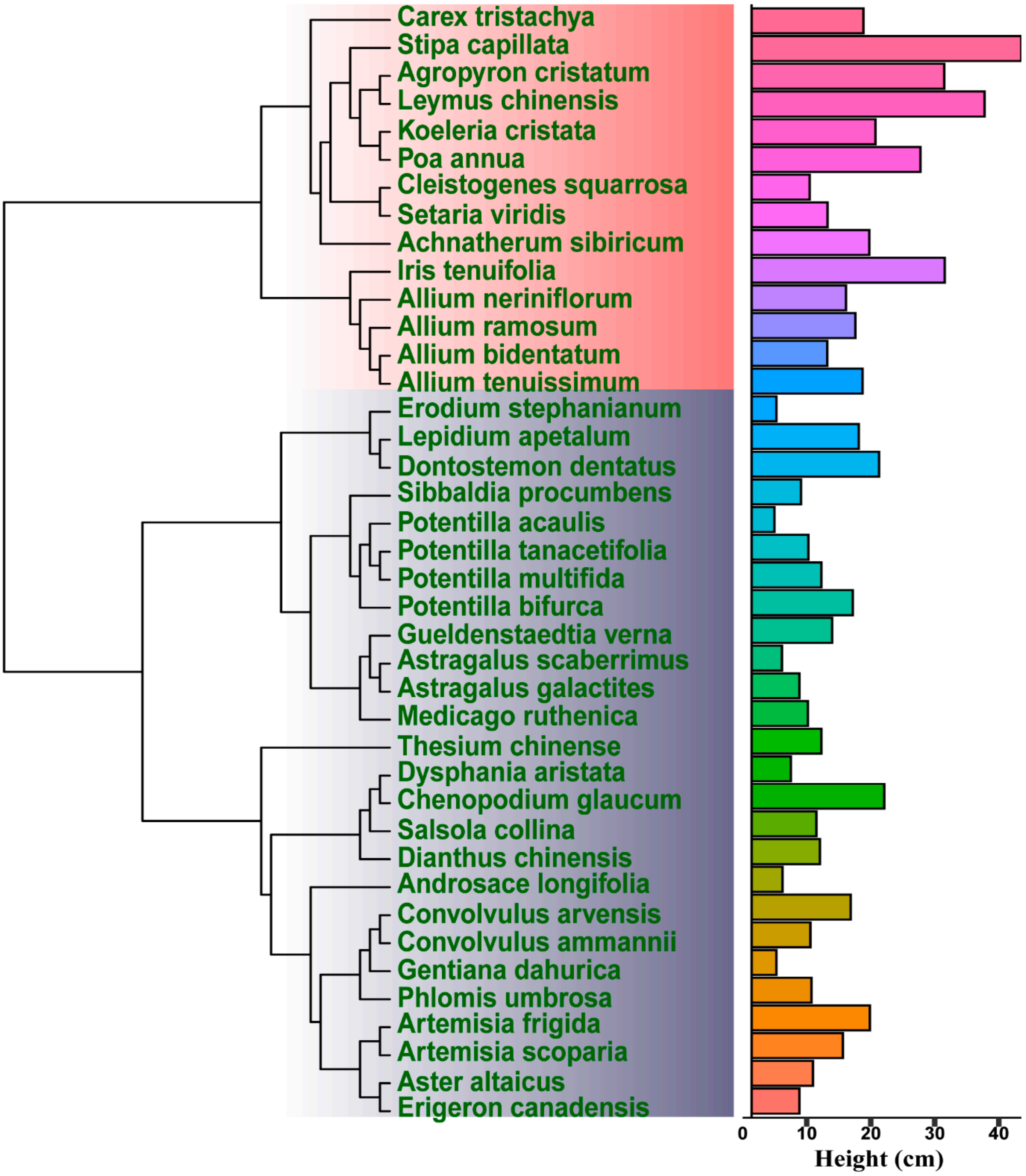

Figure S3: The distribution of plant height across the phylogeny. The weak phylogenetic signal for height (Pagel's λ =0.63, which was determined on the basis of trait values for 40 species) showed that closely related species display slightly similar in height values (i.e. exhibiting phylogenetic signals that are substantially weaker than Brownian motion expectations indicating traits were not conserved in the phylogeny).

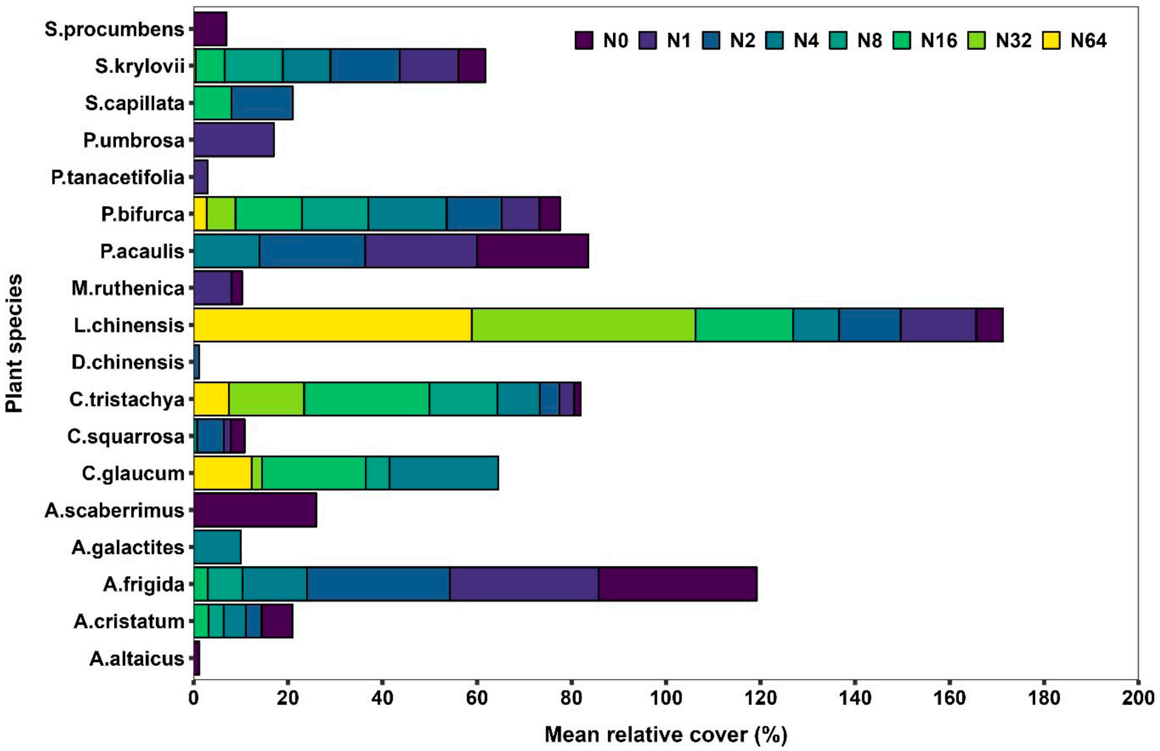

Figure S4: Top most abundant plant species based on percent relative cover ≥1% of the plants occurrence in sampling plots along the N addition gradient in the growing seasons of 2015 and 2016. N0, N1, N2, N4, N8, N16, N32, and N64 represent nitrogen fertilizer rates of 0, 1, 2, 4, 8, 16, 32, and 64 g N m^{-2} yr^{-1}, respectively.

Table S1: Most abundant plant functional group selected based on the relative cover ≥1 % (n=17).

Family name	Species	Functional group
Asteraceae	Aster altaicus	Forb
Poaceae	Agropyron cristatum	Grass
Asteraceae	Artemisia_frigida	Forb
Fabaceae	Astragalus galactites	Leguminous
Fabaceae	Astragalus scaberrimus	Forb
Amaranthaceae	Chenopodium glaucum	Forb
Poaceae	Cleistogenes squarrosa	Grass
Cyperaceae	Carex tristachya	Forb
Caryophyllaceae	Dianthus chinensis	Forb
Poaceae	Leymus chinensis	Grass
Fabaceae	Medicago ruthenica	Leguminous
Rosaceae	Potentilla acaulis	Forb
Rosaceae	Potentilla bifurca	Forb
Rosaceae	Potentilla tanacetifolia	Forb
Rosaceae	Potentilla tanacetifolia	Forb
Lamiaceae	Phlomis umbrosa	Forb
Poaceae	Stipa krylovii	Grass
Rosaceae	Sibbaldia procumbens	Forb

Table S2: Pearson's correlation result showing relationship between productivity and multidimensional diversity metrics.

**Diversity metrics**	**r^2**	***p***
T0	-0.38	<0.001
T1(Shannon)	-0.31	<0.001
T2(invers Simpson)	-0.26	<0.001
Faith's PD	-0.32	<0.001
Mean Pairwise Distance (MPD)	0.02	**0.82**
Net Relatedness Index (NRI)	-0.22	<0.01
Net Taxonomic Index (NTI)	-0.26	<0.001
FDis-Multi	-0.13	**0.07**
FDis-Height	-0.06	**0.39**
FDis-Chlorophyll	-0.10	**0.17**
CWM_{Height}	0.27	<0.001
$CWM_{Chlorophyll}$	0.26	<0.001

Insignificant correlations are presented in bold ($P > 0.05$)

The abbreviations in the above table are according to the caption of Fig. S1.

Table S3: A summary of the final generalized linear models (GLM) showing the effects of N addition on plant diversity-biomass production relationships in temperate steppe of Inner Mongolia. Significant effects (*P* < 0.05) were indicated in bold.

Terms	df	Slope	SE	z	*χ2*	*P*	*AIC*
Intercept	-	6.62	0.23	29.05	-	<0.001	183.58
SR	1	0.24	0.22	1.07	1.14	0.28	
qSR	1	-0.12	0.07	-1.56	2.43	0.12	
N	7	-	-	-	24.46	**<0.001**	
Time	5	-	-	-	73.73	**<0.001**	
Intercept	-	6.35	0.25	25.82	-	<0.001	185.10
NRI	1	0.05	0.14	0.32	0.10	0.75	
N	7	-	-	-	78.21	**<0.001**	
Time	5	-	-	-	68.31	**<0.001**	
Intercept	-	6.52	0.14	45.45	-	<0.001	185.83
FDis-Height	1	-0.48	0.43	-1.12	1.26	0.26	
qFDis-Height	1	0.46	0.42	1.10	1.21	0.27	
N	7	-	-	-	79.56	**<0.001**	
Time	5	-	-	-	71.10	**<0.001**	
Intercept	-	5.70	0.32	17.76	-	<0.001	172.16
FDis-Chlorophyll	1	-0.98	0.41	-2.38	1.20	<0.27	
FDis-Chlorophyll x N	7	-	-	-	24.67	**<0.001**	
N	7	-	-	-	70.38	**<0.001**	
Time	5	-	-	-	71.16	**<0.001**	
Intercept	-	4.81	0.73	6.57	-	<0.001	184.57
CWM_{Height}	1	1.53	0.67	2.27	2.32	0.12	
$qCWM_{Height}$	1	-0.34	0.15	-2.20	1.16	0.28	
CWM_{Height} x N	7	-	-	-	17.44	**<0.05**	
$qCWM_{Height}$ x N	7	-	-	-	15.91	**<0.05**	
N	7	-	-	-	81.15	**<0.001**	
Time	5	-	-	-	73.15	**<0.001**	
Intercept	-	3.10	0.58	5.26	-	<0.001	150.53
$CWM_{Chlorophyll}$	1	0.26	0.22	5.76	28.75	**<0.001**	
$CWM_{Chlorophyll}$ x N	5	-	-	-	40.32	**<0.001**	
N	7	-	-	-	56.23	**<0.001**	
Time	5	-	-	-	120.20	**<0.001**	
N x Time	35				76.30	**<0.001**	

SR: Species richness; qSR: second degree quadratic term of Species Richness; NRI*:* Net Relatedness Index; FDis-Height and FDis-Chlorophyll: Functional diversity of Height and Chlorophyll content, respectively; CWM_{Height} and $CWM_{Chlorophyll}$: plot level plant height and leaf chlorophyll content, respectively, values weighted by species abundance within the community; qFDis-Height and $qCWM_{Height}$: second degree quadratic term of FDis-Height and CWM_{Height},

respectively; N: various rate of Nitrogen addition effect; Time: measuring time effect; and N x Time: Interactive effects of N and measuring time.

Table S4: Analysis of deviance table for the final generalized linear mixed model **(GLMM)** included the linear and quadratic terms of diversity metrics and all possible interaction terms of N addition and measuring time effects on plant diversity-biomass production relationships to ran maximal analysis of covariance (ANCOVA).

Term	**Statistics**						
	df	*Slope*	*SE*	*t-value*	*χ2*	*P*	*AIC*
Intercept	-	6.52	0.26	25.01	-	<0.001	231.33
SR	1	0.24	0.24	0.97	0.96	0.33	
qSR	1	-0.11	0.07	-1.51	2.27	0.13	
N	7	-	-	-	17.44	**<0.05**	
Time	5	-	-	-	81.35	**<0.001**	
Intercept	-	6.31	0.25	25.21	-	<0.001	228.40
NRI	1	0.07	0.14	0.51	0.26	0.60	
N	7	-	-	-	54.15	**<0.001**	
Time	5	-	-	-	75.77	<**0.001**	
Intercept	-	6.53	0.16	41.51	-	<0.001	228.85
FDis-Height	1	-0.56	0.43	-1.29	1.68	0.19	
qFDis-Height	1	0.56	0.42	1.33	1.76	0.18	
N	7	-	-	-	49.56	**<0.001**	
Time	5	-	-	-	78.62	**<0.001**	
Intercept	-	5.74	0.32	18.05	-	<0.001	220.47
FDis-Chlorophyll	1	-0.92	0.40	-2.30	0.94	0.33	
FDis-Chlorophyll x N	7	-	-	-	22.93	**<0.01**	
N	7	-	-	-	51.42	**<0.001**	
Time	5	-	-	-	74.91	**<0.001**	
Intercept	-	4.96	0.74	6.70	-	<0.001	243.22
CWM_{Height}	1	1.35	0.70	1.97	3.08	0.07	
$qCWM_{Height}$	1	-0.29	0.15	-1.90	1.94	0.16	
CWM_{Height} x N	7	-	-	-	17.18	**<0.05**	
$qCWM_{Height}$ x N	7	-	-	-	15.76	**<0.05**	
N	7	-	-	-	66.26	**<0.001**	
Time	5	-	-	-	78.10	**<0.001**	
Intercept	-	3.17	0.60	5.50	-	<0.001	257.61
$CWM_{Chlorophyll}$	5	1.23	0.21	5.71	26.05	**<0.001**	
$CWM_{Chlorophyll}$ x N	5				41.73	**<0.001**	
N	7	-	-	-	42.43	**<0.001**	
Time	5	-	-	-	128.57	**<0.001**	
N x Time	35				81.64	**<0.001**	

Significant effects ($P < 0.05$) are indicated in bold. The abbreviations in the above table are according to the caption of Table S3.

Table S5: The Akaike Information Criterion (AIC) of generalized additive models (GAM), generalized linear models (GLM), and generalized linear mixed models (GLMM)

Terms	GAM	GLM	GLMM
SR	177.22	183.58	231.33
NRI	179.17	185.10	228.40
FDis-Height	178.80	185.83	228.85
FDis-Chlorophyll	170.20	172.16	220.47
CWM_{Height}	183.57	184.57	243.22
$CWM_{Chlorophyll}$	141.64	150.53	257.61

Diversity abbreviations are according to the caption of Table S3.
